# A genetically stable Zika virus vaccine candidate protects mice against virus infection and vertical transmission

**DOI:** 10.1038/s41541-021-00288-6

**Published:** 2021-02-17

**Authors:** Awadalkareem Adam, Camila R. Fontes-Garfias, Vanessa V. Sarathy, Yang Liu, Huanle Luo, Emily Davis, Wenqian Li, Antonio E. Muruato, Binbin Wang, Renat Ahatov, Yoseph Mahmoud, Chao Shan, Samantha R. Osman, Steven G. Widen, Alan D. T. Barrett, Pei-Yong Shi, Tian Wang

**Affiliations:** 1 Department of Microbiology & Immunology, University of Texas Medical Branch, Galveston, TX USA; 2 Department of Biochemistry & Molecular Biology, University of Texas Medical Branch, Galveston, TX USA; 3 Department of Pathology, University of Texas Medical Branch, Galveston, TX USA; 4 Sealy Institute for Vaccine Sciences, University of Texas Medical Branch, Galveston, TX USA; 5 Molecular Genomics Core Facility, University of Texas Medical Branch, Galveston, TX USA; 6 Sealy Center for Structural Biology & Molecular Biophysics, University of Texas Medical Branch, Galveston, TX USA

**Keywords:** Immunology, Microbiology, Diseases

## Abstract

Although live attenuated vaccines (LAVs) have been effective in the control of flavivirus infections, to date they have been excluded from Zika virus (ZIKV) vaccine trials due to safety concerns. We have previously reported two ZIKV mutants, each of which has a single substitution in either envelope (E) glycosylation or nonstructural (NS) 4B P36 and displays a modest reduction in mouse neurovirulence and neuroinvasiveness, respectively. Here, we generated a ZIKV mutant, ZE4B-36, which combines mutations in both E glycosylation and NS4B P36. The ZE4B-36 mutant is stable and attenuated in viral replication. Next-generation sequence analysis showed that the attenuating mutations in the E and NS4B proteins are retained during serial cell culture passages. The mutant exhibits a significant reduction in neuroinvasiveness and neurovirulence and low infectivity in mosquitoes. It induces robust ZIKV-specific memory B cell, antibody, and T cell-mediated immune responses in type I interferon receptor (IFNR) deficient mice. ZIKV-specific T cell immunity remains strong months post-vaccination in wild-type C57BL/6 (B6) mice. Vaccination with ZE4B-36 protects mice from ZIKV-induced diseases and vertical transmission. Our results suggest that combination mutations in E glycosylation and NS4B P36 contribute to a candidate LAV with significantly increased safety but retain strong immunogenicity for prevention and control of ZIKV infection.

## Introduction

Zika virus (ZIKV), is a re-emerging flavivirus of the family *Flaviviridae*, a group of single-stranded, positive-sense RNA viruses, which can be transmitted by mosquito bites, or by sexual contact^1–3^. The virus has caused more than one million human cases during the recent outbreaks in the Americas and Caribbean^4–6^ and has been associated with severe neurological diseases, such as the autoimmune disorder Guillain-Barre syndrome in adults and congenital Zika syndrome (CZS) in fetuses and infants^7,8^. Adult women of child-bearing age, in particular pregnant women, are potential risk populations for ZIKV infection. Multiple platforms have been utilized for ZIKV vaccine development, including purified inactivated virus, plasmid DNA, mRNA, adenovirus-vector, measles virus-vector, and live-attenuated vaccines, which elicit strong neutralizing antibodies and protect against ZIKV viremia in mice and/or non-human primates (NHPs)^9–12^. Despite these research efforts, no vaccines are currently approved for human use. The development of a safe, efficient vaccine with durable immunogenicity remains a high priority.

Live attenuated vaccines (LAVs) are one of the most important strategies to control flavivirus diseases. This has been demonstrated by the yellow fever virus (YFV) 17D and Japanese encephalitis virus (JEV) SA14-14-2 LAVs for controlling YFV and JEV infections, respectively. The flavivirus genome encodes a total of 10 proteins, including capsid, membrane, and envelope (E), and seven nonstructural (NS) proteins: NS1, NS2A, NS2B, NS3, NS4A, NS4B, and NS5. Among them, the NS4B protein is known to be involved in flavivirus replication and evasion of host innate immunity^13–17^. The amino acid (aa)35 to aa60 region of the N-terminal domain of NS4B protein resembles an immunomodulatory tyrosine inhibitory motif shared by many components of mammalian cell-signaling cascades^18^, which is associated with NS4B antagonist activities for antiviral innate cytokine signaling^14–16^. The NS4B-P38 is highly conserved among mosquito-borne flaviviruses, including West Nile virus (WNV). By using site-directed mutagenesis, we have previously generated single mutants of WNV and ZIKV at this site. For example, the WNV NS4B-P38G mutant had significantly reduced neuroinvasiveness^19^ but triggered stronger protective immune responses in mice than did the parent strain WT WNV NY99^20^. The mutation at the ZIKV NS4B-P36 (equivalent to WNV NS4B-P38) also led to modestly attenuated neuroinvasiveness in mice deficient in IFN-α/β and IFN-γ receptors (AG129)^21^. The envelope (E) protein of flaviviruses is responsible for cell surface receptor binding. A mutation at the E glycosylation, ZIKV E-N154Q, also caused decreased neurovirulence in mice and induction of robust innate and adaptive immunity^22^.

To increase the potential of genetic stability and ultimately the safety of either single mutant, in this study we have generated the ZE4B-36 mutant by combining both E-N154Q and NS4B-P36A mutations. Our results show the ZE4B-36 double mutant exhibits low genetic diversity upon serial passaging, reduced infectivity in mosquitoes, and significantly attenuated neurovirulence and neuroinvasiveness in mice. In addition, the mutant induces strong memory B cell and strong neutralization antibody responses, and durable T cell-mediated immunity. The mutant also protects mice from lethal WT ZIKV infection and prevents maternal to fetus transmission.

## Results

### Characterization of ZE4B-36 replication in cell culture

**Fig. 1:**
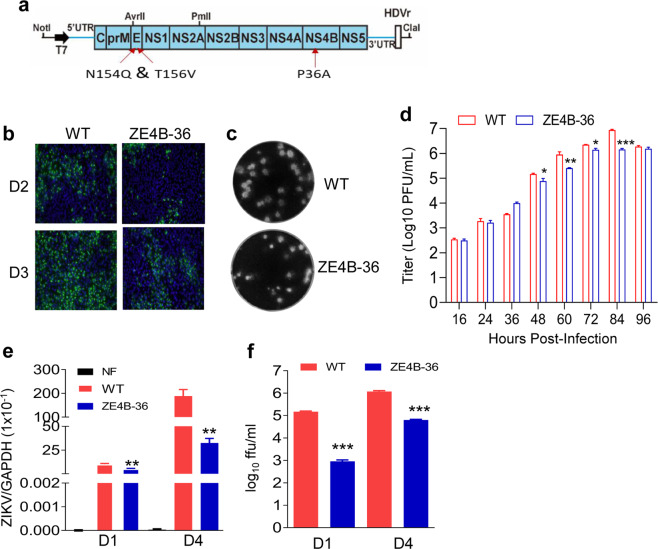
Characterization of ZE4B-36 replication in cell culture. **a** Schematic of construction of ZIKV FSS13025 mutant virus. Restriction enzyme sites used for cloning are indicated. **b** IFA of viral protein expression in Vero cells transfected with WT FSS13025ic (WT) and ZE4B-36 RNAs. Vero cells were electroporated with 10 μg genome-length WT and ZE4B-36 RNAs RNA. On days 2 and 3 post transfection, IFA was performed to examine viral E protein expression using a mouse mAb (4G2). **c** Plaque morphologies of WT and ZE4B-36 viruses. Plaques were developed on a Vero cell monolayer after 4 days of infection. **d** Comparison of growth kinetics of WT FSS13025ic and ZE4B-36 viruses in Vero cells. Cells were infected with both viruses at an MOI of 0.01. Viral titers were measured at the indicated time points using plaque assays on Vero cells, *n* = 3. Means and SDs from three independent replicates are shown. **e**, **f** A549 cells were infected with WT FSS13025ic or ZE4B-36 virus at an MOI of 0.1. At days 1 and 4 pi, viral load was measured by Q-PCR (**e**) and FFA (**f**). *n* = 4 to 8. Data are presented as means ± standard error of the mean (s.e.m). ***P < 0.001 or **P < 0.01 compared to WT group.

We previously reported that ZIKV NS4B-P36A mutant has reduced neuroinvasiveness compared to the infectious clone (ic) derived wild-type (WT) ZIKV-FSS13025ic in mice deficient in IFN-α/β and IFN-γ receptors (AG129)^21^. Another mutant, ZIKV E-N154Q, which lacks E glycosylation, was reported to induce robust innate and adaptive immunity but has modestly decreased neurovirulence in mice^22^. To increase the genetic stability with concurrent greater attenuation, here, we generated the ZE4B-36 mutant, which combines both NS4B-P36A and E-N154Q mutations. To minimize the reversion of E glycosylation, we also engineered a second mutation T156V at the glycosylation motif Asn-X-Thr (Fig. 1a). Viral antigens were detected in Vero cells transfected with WT ZIKV-FSS13025ic and ZE4B-36 mutant RNAs (Fig. 1b). Viruses derived from the transfected cells displayed similar plaque morphologies (Fig. 1c). Full genome consensus sequencing of the ZE4B-36 virus confirmed no reversions of the engineered mutations. Further, continuous passaging of the mutant virus for five rounds (3 days per round) on Vero cells did not change the engineered substitutions (data not shown). On Vero cells, the two viruses showed comparable replication kinetics at early time points, but ZE4B-36 reproducibly generated less virus than WT ZIKV-FSS13025ic at later time points except at 96 h (Fig. 1d). Following infection in interferon-producing cells, such as A549 cells, ZE4B-36 generated significantly less virus at days 1 and 4 post infection (pi) than WT ZIKV-FSS13025ic, as determined by quantitative (Q)-PCR analysis (Fig. 1e) and focus-forming assay (FFA; Fig. 1f). Overall, these results indicate that stable ZE4B-36 mutant could be produced in cell culture and the mutant displayed lower replication kinetics.

### Passaged ZE4B-36 displays genetic stability and significantly reduced neurovirulence

**Fig. 2:**
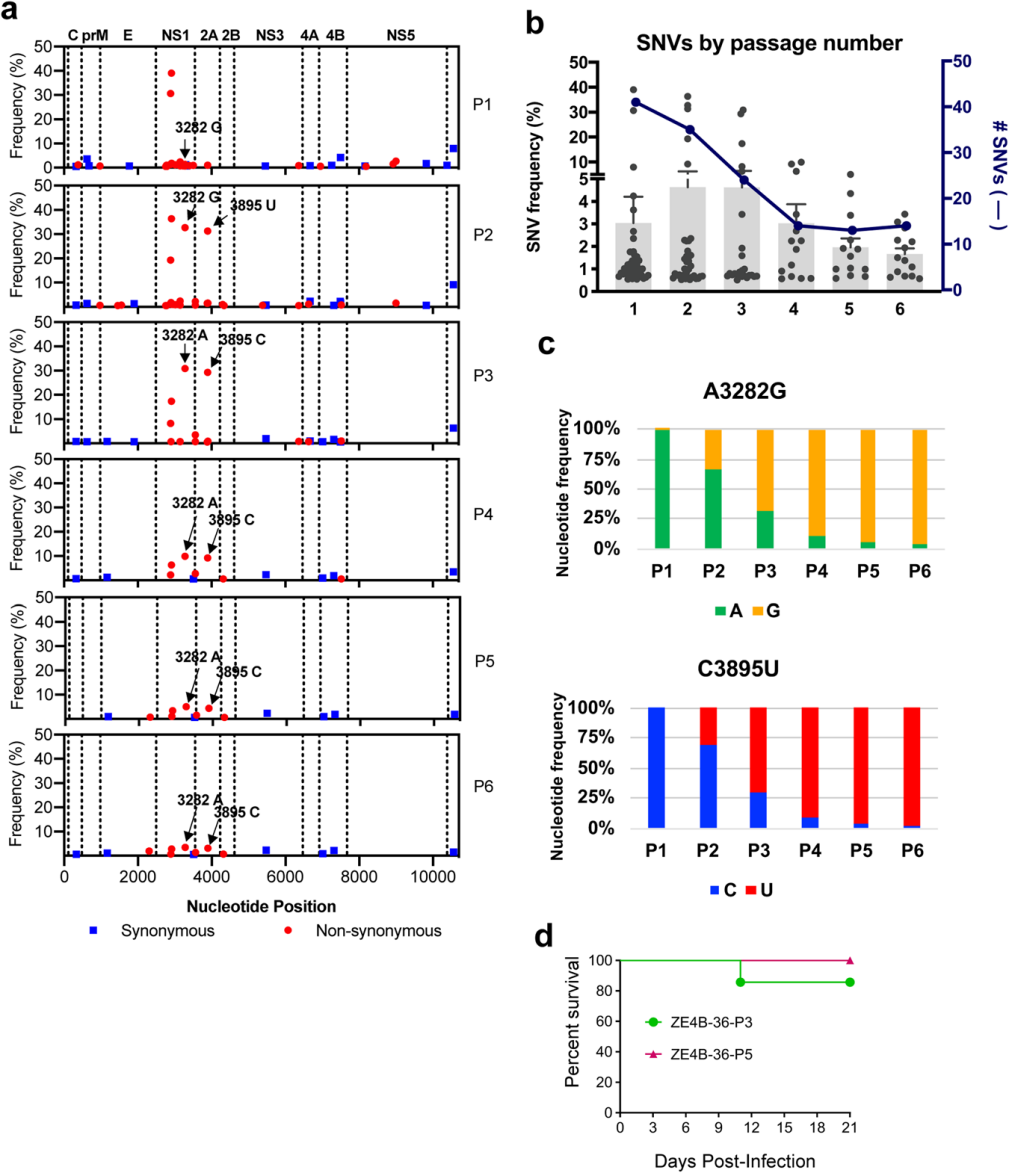
Genetic stability and neurovirulence studies of in vitro passaged ZE4B-36 mutant. **a** The genomic sequence of passage viruses was determined using NGS. Consensus sequence and sub-consensus single nucleotide variant (SNV) LofreqV2 analyses were performed for each passage. Results of SNV analysis are presented as the sub-consensus frequency of synonymous (blue squares) and non-synonymous SNVs (red circles) detected relative to position across the genome for P1–6. Arrows point to SNVs A3282G and C3895U, which increased to greater than 50% frequency at P3 becoming consensus sequence changes. Following the consensus sequence changes of 3282A to G and 3895C to U, the original nucleotides (SNVs 3282A and 3895C in P3–P6) continued to decrease in frequency**. b** Total number (blue line) and mean frequency (black symbols, mean in gray bars) of SNVs identified in each passage was determined. **c** The SNVs A3282G and C3895U and contribution of each SNV at each of the two genomic positions is presented as frequency percentage. **d** Comparison of neurovirulence between P3 and P5 of ZE4B-36 in Swiss Webster mice. One-day-old Swiss Webster mice were infected with 1.5 × 10^4^ FFU P3 (*n* = 7) and P5 (*n* = 10) of ZE4B-36 via intracranial injection. Survival percentages of infected mice are presented.

To determine the genetic stability of the ZE4B-36 mutant, we next performed next-generation sequencing (NGS) analysis of the ZE4B-36 mutant and its sequential passages in Vero cells. The consensus sequences of passage (P) 1 to P6 retained the attenuating mutations. Two additional consensus sequence changes were detected in P3 to P6: A3282G and C3895T encoding for NS1 K265E and NS2A A117V, respectively (Fig. 2a and Table 1). Both synonymous and non-synonymous single nucleotide variant (SNV)s were present throughout the genome, but only the latter ones were present at high frequencies (greater than 10%). Specifically, SNVs at 2889 and 2915 decreased from P1 to P6 (30.7% to 0.7% and 39.0% to 2.8%, respectively) indicating increased nucleotide stability at these positions. SNVs at 3282 and 3895 increased from P1 to P2, reaching 32.7% and 31.3%, respectively at P2. These SNVs at 3282 and 3895 increased to greater than 50% at P3, becoming consensus changes, and the SNVs at these positions subsequently decreased. These data indicate that the SNVs at 3282 and 3985 became more fixed in the population following passage. Further, the mean frequency of all SNVs was 3.0% at P1, then peaked at P2 and P3 with 4.6%, and decreased to 1.7% at P6 (Fig. 2b, c). The genomic sequence results indicate that passaging in Vero cells initially increased diversity, but after two passages, the sequence became more stable in both the number and frequency of SNVs.

**Table 1:** Genetic analysis of ZE4B-36 revealed the presence of four high-frequency SNVs following serial passage.

Nucleotide position	Amino Acid	Consensus codon	^a^SNV codon	^b^P1	P2	P3	P4	P5	P6
2889	NS1 V134F	GUC	UUC	30.7	19.3	8.2	2.2	1.0	0.7
2915	NS1 E142D	GAA	GAC	39.0	36.3	17.3	6.3	3.4	2.8
3282	NS1 K265E	AAA	GAA	1.4	32.8	69.1	90.1	94.9	96.6
3895	NS2A A117V	GCG	GUG	0.0	31.3	70.7	90.8	95.6	96.9

^a^SNV: single nucleotide variant with its encoding amino acid.

^b^Frequency % of each SNV for passages (P) P1–P6.

To determine the neurovirulence of the ZE4B-36 mutant, we inoculated P3 and P5 of ZE4B-36 intracranially into 1-day old Swiss Webster mice. Mice were then monitored daily for morbidity and mortality. By 3 weeks post infection, only 14.3% (1 of 7 mice) and 0% (0 of 10 mice) mortalities were observed by ZE4B-36-P3 and P5-infected mice, respectively (Fig. 2d). Furthermore, attenuating mutations and the new consensus changes at 3282 and 3895 were retained after in vivo inoculation (data not shown). Overall, NGS analysis suggests that attenuating mutations in the E and NS4B proteins are retained up to P6 and that the genetic diversity decreases following passage in Vero cells. In addition, ZE4B-36 culture passages have retained an attenuated neurovirulence phenotype.

### ZE4B-36 mutant has significantly reduced neuroinvasiveness, induces a brief infection in mice, and has attenuated infectivity in mosquitoes

**Fig. 3:**
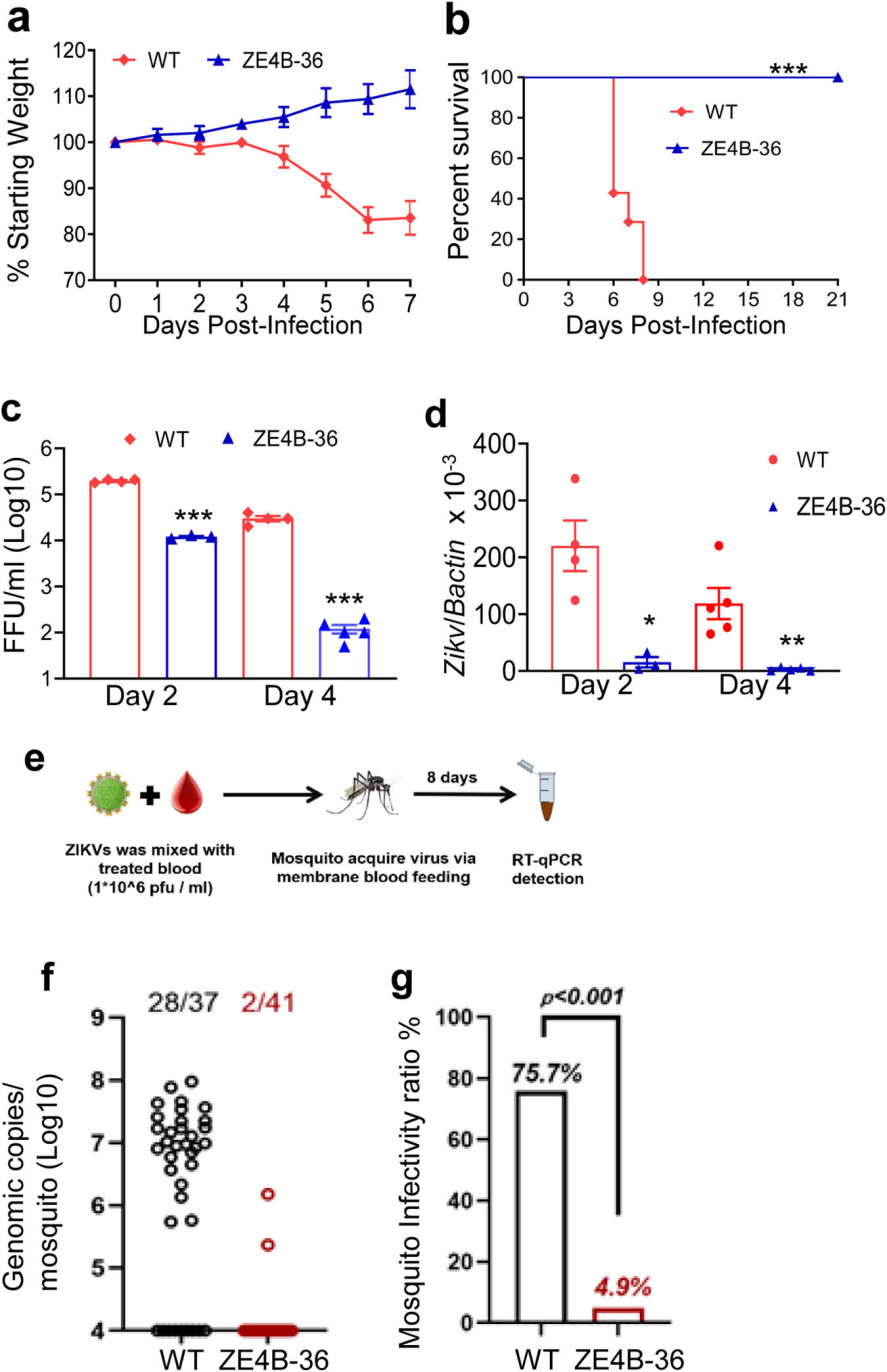
ZE4B-36 is highly attenuated in mice and mosquitoes. **a**–**d** Four-week-old AB6 mice were infected with 2.2 × 10^5^ FFU WT FSS13025ic and ZE4B-36 (*n* = 7 per group). The immunized mice were monitored for weight loss (**a**) survival (**b**). Weight loss is indicated by percentage, using the weight on the day before immunization to define 100%. ***P < 0.001 compared to the WT group (log-rank test). **c**, **d** Viremia was determined by using Q-PCR and FFA at days 2 and 4 post infection (pi). *n* = 3 to 5. Data are presented as means ± standard error of the mean (s.e.m). *** *P* < 0.001 or **P < 0.01 compared to the WT group. **e**–**g** Schematic diagram of mosquito membrane blood feeding (**e**). Infection of WT and ZE4B-36 in *Aedes aegypti*. Complement-inactivated sheep blood was inoculated with 1 × 10^6^ FFU/ml of WT ZIKV-FSS13025 and ZE4B-36 viruses. The mosquitoes were fed on the virus and blood mixture. Fully engorged mosquitoes were selected and reared for additional 8 days. Individual engorged, incubated mosquitoes were homogenized, and viral burden of each mosquito was assayed by Q-PCR and calculated into genomic copies (**f**). One dot represents one mosquito. The numbers of infected mosquitoes versus total mosquitoes are shown above each column. The threshold for distinguishing positive mosquitoes was 1 × 10^4^ genomic copies. Data were pooled from two independent biological replicates. **g** The infection ratio of each group was counted using the numbers of infected mosquitoes divided by the number of total mosquitoes. ***P < 0.001 compared to the WT group.

To characterize the attenuation phenotype of the double mutant, 4- to 6-week-old C57BL/6 mice deficient of IFN-α/β receptor (AB6) were inoculated intraperitoneally (i.p.) with 2.2 × 10^5^ FFU of the ZE4B-36 mutant. Mice injected with the same dose of the parental WT ZIKV-FSS13025ic were included as controls. All WT ZIKVFSS13025ic-infected mice exhibited significant weight loss within 1 week and succumbed to infection by day 8. In contrast, ZE4B-36-infected mice displayed neither weight loss nor any clinical signs (Fig. 3a, b). We further characterized the attenuated phenotype of ZE4B-36. Based on our prior studies with other ZIKV NS4B mutants^21^, a lower dose (1.0 × 10^4^ FFU) of ZE4B-36 or WT FSS13025ic and PBS (mock) were next inoculated into AB6 mice of similar ages. WT ZIKV-FSS13025ic-infected mice developed clinical symptoms (progressive weight loss and neurologic signs) and approximately 60% required euthanasia within two weeks. Neither the mock group nor the ZE4B-36- infected mice showed any disease signs (Supplementary Fig. 1a, b). The ZE4B-36 mutant induced lower viremia compared to the WT ZIKV-FSS13025ic group on days 2 and 4 post infection (pi) with a dose of 2.2 × 10^5^ FFU in AB6 mice (Fig. 3c, d). And at day 6 pi, viral loads in blood, uterus, testes, and brains of ZE4B-36 vaccinated-AB6 mice were barely detectable (Supplementary Fig. 1c–f). Compared to WT ZIKV-FSS13025ic infection, ZE4B-36-infected AB6 mice displayed lower levels, but similar kinetics of viremia. Overall, the ZE4B-36 mutant is attenuated in AB6 mice.

Mosquitoes acquiring flaviviruses via a blood meal from an infected host represent an essential process in the flavivirus life cycle. We evaluated the infectivity of ZE4B-36 mutant in mosquitoes. Hemotek membrane blood-feeding system was used in vitro to mimic natural virus acquiring by mosquitoes. Inactivated sheep blood was inoculated with equal titers of WT ZIKV-FSS13025ic or ZE4B-36 mutant. Starved female *Aedes aegypti* mosquitoes were allowed to feed on the reservoir containing the virus and blood mixture. Fully engorged mosquitoes were selected and reared for 8 days until quantitative polymerase chain reaction (Q-PCR) analysis (Fig. 3e). Compared to the WT ZIKVFSS13025ic, the ZE4B-36 mutant-infected mosquitoes had an extremely low viral load and infectivity ratio (Fig. 3f, g). These results suggest that the ZE4B-36 mutant was largely attenuated in the mosquitoes and had a lower ability to potentially replicate in mosquitoes. Future work will be performed to determine whether infected mosquitoes can transmit the attenuated virus and that no mutations or reversions to WT occur with replication in the mosquito.

### ZE4B-36 mutant triggers strong memory B cell and antibody responses, and durable T-cell immunity in mice

**Fig. 4:**
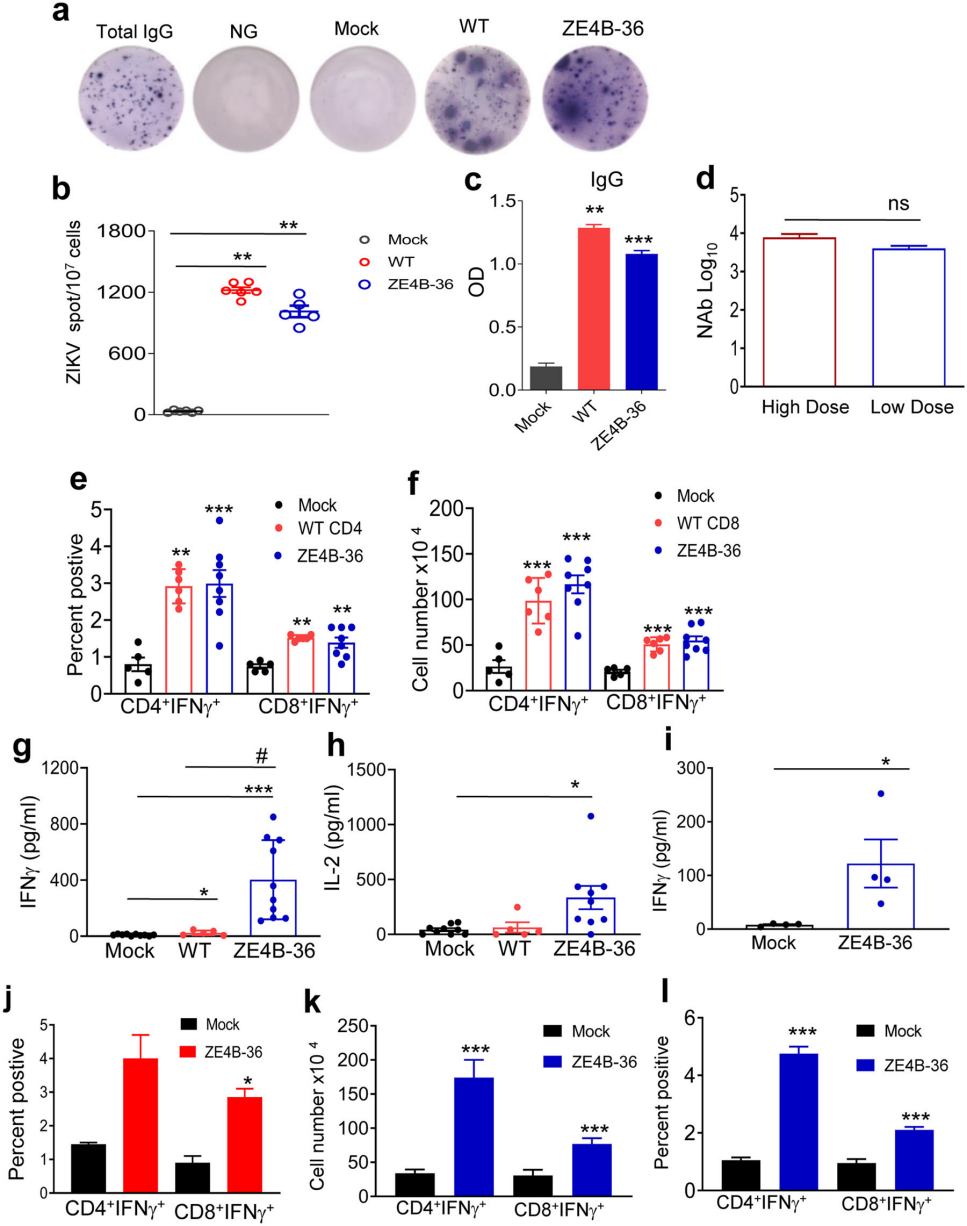
ZE4B-36 induces potent B and T cell responses. **a**–**j** Four- to 6-week old AB6 mice (**a**–**i**) or WT B6 (**j**) mice were infected with ZE4B-36, WT ZIKV FSS13025ic, or PBS (mock). On day 28 pi, splenocytes and blood were harvested. **a** Splenocytes were stimulated in vitro for 7 d with R848 plus rIL-2 and seeded onto ELISpot plates coated with Ig capture Ab or ZIKV- VLP. Images of total IgG-secreting, ZIKV-specific MBCs, and control wells are shown. **b** Frequencies of ZIKV antibody-secreting cells per 10^7^ input cells. **c** Sera ZIKV IgG antibody detected by ELISA. *n* = 4 to 6. **d** Sera NAb titers of mice vaccinated with 2.2 × 10^5^ FFU (high dose) or 1 × 10^4^ FFU ZE4B-36 (low dose). *n* = 4 to 5. Splenocytes of AB6 mice (**e**, **f**) or WT B6 mice (**j**) were cultured ex vivo with WT ZIKV for 24 h and stained for IFN-γ, CD3, and CD4 or CD8. Percentage (**e**) and the total number of IFN-γ^+^ (**f**, **j**) T-cell subsets is shown. *n* = 4 to 7. **g**–**i** Cytokine production in the ex vivo culture following stimulation with WT-ZIKV for 48 h. *n* = 4 to 10. **k**, **l** Four-week-old WT B6 mice were infected with ZE4B-36 or PBS (mock). On day 60 pi, splenocytes were cultured with WT ZIKV for 24 h and stained for IFN-γ, CD3, and CD4 or CD8. Cell number (**k**) and percent positive (**l**) of IFN-γ^+^ T-cell subsets is shown. *n* = 4. Data are presented as means ± standard error of the mean (s.e.m). ***P < 0.001, **P < 0.01, or *P < 0.05 compared to mock group.

To study its immunogenicity, AB6 mice were immunized i.p. with 1 × 10^4^ FFU of ZE4B-36 and control WT ZIKV-FSS13025ic. On day 28, both ZE4B-36 and WT ZIKV-FSS13025ic triggered strong ZIKV-specific memory B cell responses as analyzed using a conventional ELISpot assay (Fig. 4a, b). ZIKV virus-like particle (VLP)-specific IgG responses were induced in both groups (Fig. 4c). Interestingly, ZE4B-36 induced similar titers of neutralization antibodies at either low dose (1 × 10^4^) or high dose (2.2 × 10^5^, Fig. 4d). To study T cell responses, splenocytes of vaccinated mice were restimulated with WT ZIKV antigens in vitro, and ZIKV-specific T cell responses were analyzed using an intracellular cytokine staining (ICS) assay. At day 28 pi, the number and percent of ZIKV specific CD4^+^IFN-γ^+^ and CD8^+^IFN-γ^+^ T cells in the ZE4B-36 and WT ZIKV-FSS13025ic-vaccinated mice were significantly higher than those of the mock group. Notably, ZE4B-36-infected mice showed a trend of more ZIKV-specific CD4^+^IFN-γ^+^ splenocytes than WT ZIKV-FSS13025ic did (Fig. 4e, f). Furthermore, splenocytes of ZE4B-36-vaccinated mice produced significantly more IFN-γ and a trend of higher IL-2 production than those of WT ZIKV-FSS13025ic group upon in vitro stimulation with ZIKV antigens (Fig. 4g, h). A single dose subcutaneous (s.c.) vaccination with 10^4^ FFU ZE4B-36 in WT B6 mice also triggered ZIKV-specific CD4^+^IFN-γ^+^ and CD8^+^IFN-γ^+^ T cell responses and Th1 type cytokine at day 28 pi (Fig. 4i, j). ZIKV specific CD4^+^IFN-γ^+^ and CD8^+^IFN-γ^+^ T cell responses remained high at 2 months post s.c. vaccination in WT B6 mice (Fig. 4k, l), which suggests that the induction of robust T cell-mediated immune responses is independent of type I IFN signaling and the route of immunization. Overall, these results suggest that ZE4B-36 induces strong B cell and antibody immune responses, and retains durable T cell immunity in vaccinated mice.

### Vaccination with the ZE4B-36 mutant protects mice from subsequent lethal WT ZIKV challenge and prevents maternal transmission to the fetus

**Fig. 5:**
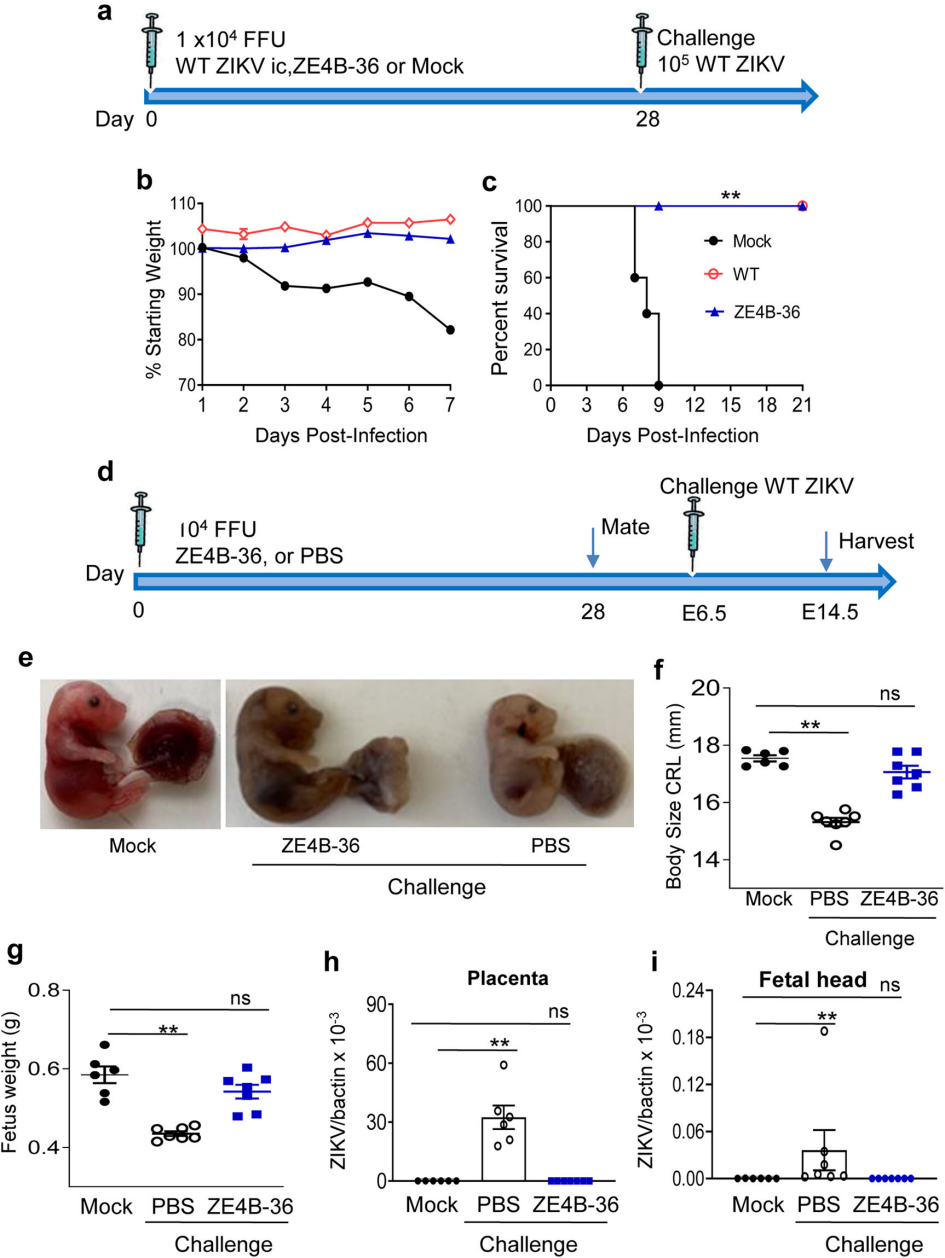
Vaccination with ZE4B-36 protects mice from subsequent WT ZIKV challenge and maternal to fetus transmission. **a**–**c** Four- to 6-week-old AB6 mice were infected i.p. with 1 × 10^4^ FFU WT ZIKV FSS13025ic, ZE4B-36 or PBS (mock). On day 28, all surviving mice were challenged with 1 × 10^5^ FFU WT ZIKV FSS13025. **a** Schematic of active vaccination and challenge. **b** Mouse weight loss. **c** Survival rate. Weight loss is indicated by percentage, using the weight on the day before immunization to define 100%. *n* = 5, 4, and 10 for PBS, WT, and ZE4B-36-infected mice, respectively. **d**–**i** Female AB6 mice were immunized with 10^4^ FFU of ZE4B-36 or PBS. At day 28 post immunization, vaccinated female mice were mated with AB6 males. Pregnant mice were challenged with 5 × 10^5^ PFU ZIKV-PRV strain at E6.5. On E14.5, animals were euthanized and fetal size, eight and viral loads were measured. Mock group represents pregnant mice without vaccination and challenge. **d** Experimental schematic. **e** Representative image of E14.5 fetuses. The size (**f**) and weight (**g**) of 7 fetuses at E14.5. **h**, **i** Viral load in placentae and fetal heads collected from 6 to 7 fetuses per group. Data are presented as means ± standard error of the mean (s.e.m). **P < 0.01 compared to mock group (unpaired *t* test).

To determine the protective efficacy of the mutant, 4-week-old AB6 mice were vaccinated with 1 × 10^4^ FFU of ZE4B-36. Mice immunized with PBS (mock) and the same dose of WT ZIKV-FSS13025ic were used as controls (Fig. 5a). At day 28 pi, all surviving mice were challenged with 1 × 10^5^ FFU WT ZIKV-FSS13025. All mice vaccinated with either WT ZIKV-FSS13025ic or ZE4B-36 survived WT ZIKV challenge and displayed neither clinical signs nor weight loss. In contrast, all mice from the mock group showed signs of disease and weight loss and succumbed to infection within a 9-day period (Fig. 5b, c). Similar studies were performed in WT B6 mice. It was noted that at day 3 post WT ZIKV challenge, viremia levels dropped more than 97% in ZE4B-36 vaccinated mice compared to the mock group (Supplementary Fig. 2a, b).

ZIKV induces CZS in fetuses and infants^7,8^. To determine the effects in protecting the host from maternal infection and maternal-to-fetal transmission during pregnancy, we immunized female AB6 mice with 1 × 10^4^ FFU of ZE4B-36 or PBS. Vaccinated female mice were mated with AB6 males at day 28. Pregnant mice were challenged with 5 × 10^5^ FFU of the mouse-adapted ZIKV Dakar 41519 strain on an embryonic day (E)6.5. Pregnant mice with neither vaccination nor challenge were used as a mock group. On E14.5, individual fetuses were weighed and the size was measured. PBS-vaccinated group displayed significantly reduced fetal weight and smaller size compared to the mock group; while no differences were detected between the ZE4B-36 group and the mock group (Fig. 5e–g). There were higher viral loads in the placentae and fetal heads of the PBS-vaccinated group compared to the mock group, but no differences were observed in the ZE4B-36 group (Fig. 5h, i). Overall, these results suggest that ZE4B-36 protects mice from ZIKV-induced severe diseases and maternal-to-fetal transmission during pregnancy.

## Discussion

The goal of this study is to develop safe and effective ZIKV vaccines for maternal immunization. Maternal vaccination can provide protection against infections for the mother, developing fetus, and the newborn through maternal antibodies^23^. Clinically, maternal vaccination has been successful in preventing influenza, tetanus, and pertussis in pregnant women and their infants^24^. For example, the inactivated influenza vaccine was administered to pregnant women in Bangladesh, and limited influenza illness by 63% in infants up to 6 months of age and prevented about one-third of all febrile respiratory illnesses in mothers and young infants^25^. Compared with other vaccine platforms currently under development for ZIKV vaccines, the LAV approach has the advantage of single-dose, quick immunity, durable protection, and low cost. Animal model studies have shown that vaccination of female mice with a ZIKV LAV before and during pregnancy prevents maternal-to-fetal transmission^12,26^. Furthermore, for infectious diseases like ZIKV in developing countries, it is essential to have vaccines with a single-dose efficacy and long-lasting immune protection. Multi-dose vaccines are practically impossible to implement in remote areas. However, for LAVs, like the YFV17D vaccine, the World Health Organization recommends immunization in pregnant women during outbreaks in endemic regions or when the risk of viral infection is high^27,28^. Similarly, wider acceptance of maternal immunization of vaccines, such as ZIKV vaccine candidates, has been hampered due to limited safety and efficacy data^29^. ZIKV vaccine trials have thus excluded pregnant women due to safety concerns^30^. In this study, we have demonstrated that the ZE4B-36 mutant has low genetic diversity upon serial passaging, significantly attenuated neurovirulence and neuroinvasiveness in mice, and low infectivity in mosquitoes, which will together contribute to the safety of the candidate vaccine.

A major challenge to the generation of safe LAVs to protect against RNA viral diseases is the inherent instability of the RNA genome^31,32^. Previous studies evaluating genomics of WT or attenuated ZIKVs found that the NS1 gene has heightened diversity^33–35^, and the present analysis found that three of the four high-frequency SNVs were in NS1: G2889U, A2915C, and A3282G. Interestingly, SNVs in the codons for amino acids NS1 265 and NS2A 117 have been detected in WT ZIKVs^33–35^, though at lower frequency; however, the NS1 K265E amino acid change was previously characterized as a Vero adaptation of WT ZIKV leading to increased replication in cells^36^. Ultimately, a dramatic decrease in the number and frequency of variants lowered the genetic diversity following passaging in Vero cells, indicating that the vaccine becomes genetically stable, i.e., the virus adapts to Vero cells. This is made even more evident by the fact that the P3 of ZE4B-36 caused 20% mortality (1 of 7 mice) in suckling mice, whereas P5 inoculation did not cause any death or signs of disease. Together the data show that the more genetically stable and phenotypically safe vaccine virus is a higher passage (P5–P6). ZE4B-36 had SNVs that became consensus changes at P3 and continued to comprise a larger proportion of the population at those positions. The incorporation of substitutions and subsequent decrease of SNVs stabilized the genetic population from P3 to P5 of the “new” ZE4B-36 virus and increased attenuation in mice. Future evaluation on the stability of ZE4B-36 mutant in tissue culture at higher passage levels is warranted and passage together with WT strain to ensure the mutations are not “lost”.

ZIKV NS4B-P36 single mutant has previously shown to have a modestly attenuated neuroinvasiveness in AG129 mice^21^. Here, we demonstrated that ZE4B-36 has highly attenuated neuroivasiveness in mice similar to the ZIKV E-N154Q single mutant^22^. We also found that 1-day old Swiss Webster mice all survived an i.c. infection with a dose more than 10^4^ FFU of ZE4B-36 P5 and less than 20% mortality rate with the same dose of ZE4B-36 P3. YFV 17D vaccine is generally considered one of the safest and most effective LAVs ever developed. It was previously shown that 3-week-old mice inoculated intracerebrally with 10^3.5^ PFU YFV17D displayed 100% lethality^37^, indicating the neurovirulence of the vaccine. Thus, ZE4B-36 has significantly reduced neurovirulence compared to YFV 17D. Our prior study also suggests that knockout of E glycosylation did not significantly affect neurovirulence in the ZIKV E-N154Q mutant^22^. Overall, unlike the E or NS4B single mutants, the ZE4B-36 double mutant is highly attenuated in both neuroivasiveness, and neurovirulence in mice.

The ZIKV E-N154Q was reported to have diminished oral infectivity for the *A. aegypti* vector^22^. In comparison, NS4B mutations in flaviviruses, such as WNV NS4B P38G, result in increased vector competence compared to WT viruses in mosquitoes^38^. We also found that that ZE4B-36 has reduced infectivity in *Aedes* mosquitoes, which may result from the single mutation of E-N154Q^22^. The reduced ability to infect mosquito vector may add to the safety profile of the live attenuated ZE4B-36 vaccine candidate.

ZIKV E-N154Q is also known to induce strong neutralization antibodies following vaccination and protects mice from ZIKV infection^22^. Here, we demonstrated that ZE4B-36 induced robust ZIKV specific memory B cell, and neutralization antibody responses. T cells play a central role in adaptive immunity and have been shown to contribute to host protection against several flaviviruses infections. They are directly involved in viral clearance and/or provide help for B cells and antibody maturation^39–43^. Studies in animal models suggest that T cells are important for ZIKV clearance and host protection^44,45^. Following a single dose injection, ZE4B-36 induces strong ZIKV-specific CD4^+^ and CD8^+^ T cell responses in both WT B6 mice and AB6 mice. Notably, the responses remain high more than 2 months post vaccination. In summary, ZE4B-36 retains strong immunogenicity like the NS4B and E single mutants.

One major issue with flavivirus vaccines is the potential ability to induce infection enhancing antibodies. Cross-reactive DENV antibodies have been reported to have antibody-dependent enhancement (ADE) effects^46,47^. Administration of DENV- or WNV-convalescent plasma into ZIKV-susceptible mice also resulted in increased morbidity (including fever, viremia, and viral loads in spinal cord and testes) and mortality^48^. Future investigation will need to test the cross-neutralizing potential or ADE effects of the antibodies generated in ZE4B-36- vaccinated mice. ZIKV has been associated with CZS in fetuses and infants, including microcephaly, and intrauterine growth restriction^7,8^. Although the number of human cases of ZIKV infection has dropped in most countries since 2017, the virus has spread widely and unpredictably. Compared to other emerging arboviruses, ZIKV is unique in the breadth of serious complications (such as the devastating CZS) it can cause. The virus has become endemic in the Americas and is likely to cause sporadic cases and localized outbreaks in the future. Thus, a vaccine to protect from CZS, particularly for the potential risk population is still urgently needed and should be pursued as a public health priority^49,50^. Our studies here suggest that the ZE4B-36, which combines mutations in both NS4B P36 and E glycosylation sites, elicits and maintains strong immunogenicity as the E or NS4B single mutants and other LAVs do, but demonstrates significantly improved safety by attenuation of neuroinvasiveness and neurovirulence, reduction of genetic diversity upon serial culture passages, and decreasing infectivity in mosquitoes. Together, these properties make ZE4B-36 an attractive LAV candidate.

## Methods

### Viruses

ZIKV FSS13025 strain (ZIKV-FSS13025)^51^, ZIKV PRVABC59 (ZIKV-PRV) strain, and ZIKV Dakar strain 41525 (ZIKV-Dakar) were obtained from the World Reference Center for Emerging Viruses and Arboviruses (WRCEVA) at the University of Texas Medical Branch (UTMB) and were amplified once or twice in Vero cells. Standard molecular biology procedures were performed for the plasmid construction of the ZE4B-36 mutant. The NS4B P36A mutations were introduced to the ZIKV Env glycosylation mutant cDNA infectious clone pFLZIKV^22^. Overlapping PCR assays containing the mutation were performed to amplify the DNA fragment between unique restriction sites^52^. Full-length plasmids were validated by DNA sequencing. All primers are listed in Table 2. Virus RNA of the complete genome of ZIKV-FSS13025 and ZE4B-36 was in vitro transcribed using a T7 mMessage mMachine kit (Ambion, Austin, TX) from cDNA plasmids pre-linearized by ClaI. The RNA was precipitated with lithium chloride, washed with 70% ethanol, resuspended in RNase-free water, quantitated by spectrophotometry, and stored at −80 °C in aliquots. The RNA transcripts (10 µg) were electroporated into Vero cells following a protocol described previously^52,53^. ZE4B-36 mutant was passaged sequentially in Vero cells six times. Unless specified, most vaccination studies described here used passage 5 of ZE4B-36 mutant.

**Table 2:** PCR Primers used in the construction of ZE4B-36 mutant.

Primer	Sequence (5′–3′)
Zika-6138V	CGACCTGAGGCCGACAAAGTAGCAGC
Zika-8470R	CTTACCACAGCCCGCGTGCCAG
NS4B-P36A-F	GACA TTGACCTGCGGGCCGCCTCAGCTTGGGCT
NS4B-P36A-R	AGCCCAAGCTGAGGCGGCCCGCAGGTCAA TGTC
E-N154Q + T156V-F	AGTGGGATGATCGTTCAGGATGTTGGACATGAA
E-N154Q + T156V-R	TTCATGTCCAACATCCTGAACGATCATCCCACT

### Mice

Four- to six-week-old C57BL/6 (B6) mice and B6 mice deficient in the IFN-α/β receptor (AB6) were bred and maintained at UTMB. Mice were inoculated intraperitoneally (i.p.) or subcutaneous injection (s.c) with 1 × 10^4^ or 2.2 × 10^5^ focus forming unit (FFU) of the ZE4B-36 mutant (Vero cell passage 5) or the WT ZIKV-FSS13025ic. In some experiments, mice were rechallenged with 1 × 10^5^ FFU of WT ZIKV-FSS13025 at day 28 post primary infection. In addition, vaccinated mice were set up for timed-mating and were i.p. inoculated with 5 × 10^5^ plaque-forming unit (PFU) ZIKV-PRV strain at E 6.5. Infected mice were monitored twice daily for signs of morbidity. On E14.5, mice were euthanized. Fetus weight and size were measured. Placentae and fetuses were collected for viral load studies. For neurovirulence studies, timed pregnant Swiss Webster mice were ordered from Taconic (Rensselaer, NY). Vaccinated WT B6 mice were pretreated with 1 mg/ mouse of mouse MAR1-5A3 i.p. followed by infection with 1 × 10^5^ FFU ZIKV–Dakar–MA strain 1 day later. For neurovirulence study, groups of 1 day old outbred Swiss Webster mice were injected with ZE4B-36 passaged viruses with 1.5 × 10^4^ FFU by the intracranial (i.c.) route. Mice were monitored daily for morbidity and mortality. All animal experiments were approved by the Animal Care and Use Committee at UTMB.

### ZIKV infection in cell culture

Vero (4 × 10^5^ cells/well) cells were seeded into a 12-well plate one day prior to infection. At 24 h post seeding, cells were infected with ZE4B-36 or WT ZIKV-FSS13025ic at a multiplicity of infection (MOI) of 0.01. Each infection was performed in triplicate. After incubation at 37 °C (Vero) for 1 h, cells were washed extensively with phosphate-buffered saline (PBS) to eliminate the unbound virus. One milli liter of fresh medium was then added to each well. From day 1 to day 5 p.i., supernatants were collected daily and clarified by centrifugation prior to storage at −80 °C. Virus titers were determined using a plaque assay. A549 cells grown in 12-well plates were infected with an MOI of 0.1 of either ZIKV-FSS13025ic or ZE4B-36. After incubating the viruses with the cells for 1 h at 37 °C, cells were washed, and MEM containing 2% FBS was added to the cells. The plates were incubated at 37 °C with 5% CO_2_. On days 1 and 4 p.i., culture supernatants and cells were collected to measure viral loads.

### NGS analysis

ZE4B-36 was passaged in Vero cells and sequences obtained using NGS as previously described^33^. Briefly, RNA was extracted from the virus in culture supernatants using the QIAamp Viral RNA Mini kit (Qiagen). cDNA libraries were generated with random hexamers using the TruSeq RNA v2 kit (Illumina) and sequenced using Illumina NextSeq 550. Paired-end reads were processed using open source packages, and the pipeline is described previously^34^. Sequencing data are available in the ArrayExpress database (accession E-MTAB-9496). To study the genetic diversity of the genomic sequences, SNV analysis was performed using the sensitive SNV caller LoFreq version 2^54^. Data were exported to MS Excel version 16 for sorting, and frequencies were plotted and visualized using GraphPad Prism version 8. To determine genetic stability in vivo, virus sequencing from mouse brains was performed. RNA was harvested from dead suckling mouse brain homogenates. RNA was amplified using RT-PCR (Roche Titan One Tube RT-PCR kit) with sequence-specific primers, and amplicons were subjected to Sanger sequencing in order to obtain sequence information for the engineered mutations and the areas of high genomic diversity.

### ZIKV infection in mosquitoes

*A. aegypti* Rockefeller mosquitoes were reared in the illuminated incubator (Model 818, Thermo Fisher Scientific) at 28 °C, 80% humidity, and 12 h light following the standard rearing procedures. Seven-day old female mosquitoes were separated into mesh-covered cartons and fed with cotton balls containing 10% sucrose. Complement-inactivated sheep blood (C26218, Hemostat laboratories) was inoculated with 1 × 10^6^ FFU/ml of WT ZIKV-FSS13025 and ZE4B-36 mutant viruses, respectively. The mosquitoes were first starved for 24 h by removing the cotton balls from the cartons and then fed on the virus and blood mixture using the Hemotek system (5W1, Hemotek limited). Fully engorged mosquitoes were selected and reared for additional 8 days. Live mosquitoes were collected for RNA extraction and Q-PCR assays were performed to determine viral loads.

### FFA for viral titer

Vero cell monolayers were initially incubated with sample dilutions for 1 h. A semi-solid overlay containing 0.8% methylcellulose (Sigma-Aldrich), 3% fetal bovine serum (FBS), 1% penicillin–streptomycin, and 1% l-glutamine (Invitrogen) was then added. At 48 h, the overlay was removed, cell monolayers were washed, air dried, and fixed with 1:1 of acetone: methanol solution for at least 30 min at −20 °C. Cells were next subjected to immunohistochemical staining with a ZIKV hyperimmune mouse ascitic fluid antibody (T-36846, World Reference Center for Emerging Viruses and Arboviruses, WRCEVA, Galveston, TX) followed by goat anti-mouse peroxidase-conjugated IgG (SeraCare Life Science, Inc, Milford, MA) at room temperature for 1 h. Cells were incubated with a peroxidase substrate (Vector Laboratories, Burlingame, CA) until color developed. The number of foci was counted and used to calculate viral titers expressed as FFU/ml.

### Quantitative PCR

Viral-infected cells or tissues were resuspended in Trizol (Invitrogen) for RNA extraction. Complementary (c) DNA was synthesized by using a qScript cDNA synthesis kit (Bio-Rad, Hercules, CA). The sequences of the primer sets for ZIKV and PCR reaction conditions were described previously^55^. The PCR assay was performed in the CFX96 real-time PCR system (Bio-Rad). Gene expression was calculated using the formula $2^{-[{\mathrm{C}}_{{\mathrm{t}}}({\mathrm{target}}\,{\mathrm{gene}})-{\mathrm{C}}_{{\mathrm{t}}}({\beta}-{\mathrm{actin}})]}$ as described before^56^.

### Intracellular cytokine staining (ICS)

Splenocytes (2.5 × 10^6^) were incubated with 0.1 × 10^6^ FFU live ZIKV-FSS13025 for 24 h. BD GolgiPlug (BD Bioscience) was added to block protein transport at the final 6 h of incubation. Cells were stained with antibodies for CD3, CD4, or CD8 fixed in 2% paraformaldehyde and permeabilized with 0.5% saponin before adding anti-IFN-γ, or control rat IgG1 (e-Biosciences). Samples were processed with a C6 Flow Cytometer instrument. Dead cells were excluded on the basis of forwarding and side light scatter. Data were analyzed with a CFlow Plus Flow Cytometer (BD Biosciences).

### Immunofluorescence assay (IFA)

IFA was performed according to a previously described protocol^57^. Briefly, Vero cells transfected with viral RNA were grown in an 8-well Lab-Tek chamber slide (Thermo Fisher Scientific, Waltham, MA). At various time points, the cells were fixed in 100% methanol at −20 °C for 15 min. After 1 h incubation in a blocking buffer containing 1% FBS and 0.05% Tween-20 in PBS, the cells were treated with a mouse monoclonal antibody 4G2 for 1 h and washed 3 times with PBS (5 min for each wash). The cells were then incubated with Alexa Fluor^®^ 488 goat anti-mouse IgG for 1 h in blocking buffer, after which the cells were washed three times with PBS. The cells were mounted in a mounting medium with DAPI (4′,6-diamidino-2-phenylindole; Vector Laboratories, Inc.). Fluorescence images were observed under a fluorescence microscope equipped with a video documentation system (Olympus). The images were processed using ImageJ software (National Institutes of Health, MD).

### ELISA

Serum samples collected at day 28 post infection were tested for ZIKV IgG and IgM antibodies by using ELISA assays. ELISA plates were coated with 50 ng/well recombinant ZIKV-E protein (Bioscience) for overnight at 4 °C. The plates were washed twice with PBS, containing 0.05% Tween-20 (PBS-T) and then blocked with 8% FBS for 2.5 h. Sera were diluted at 1:40 in blocking buffer and 100 µl was added per well for 1 h at RT. Plates were washed five times with PBS-T. Goat anti-mouse IgG (Sigma, MO, USA) coupled to alkaline phosphatase was added as the secondary antibody at a 1:1000 dilution for one hour at RT. The color was developed with *p*-nitrophenyl phosphate (Sigma-Aldrich) and the intensity was read at an absorbance of 405 nm.

### ELISPOT assay

ELISpot assays were performed as previously described^58^ with some modifications. Briefly, splenocytes were stimulated with 1 µg/ml R848 and 10 ng/ml recombinant human IL-2 (purchased from Mabtech In, OH). Millipore ELISPOT plates (Millipore Ltd, Darmstadt, Germany) were coated with ZIKV VLP (The Native Antigen Company, Oxford, UK, 15 mg/ml). To detect total IgG, the wells were coated with anti-human Ig capture Ab (Mabtech In). The stimulated splenocytes were harvested, washed, and added in duplicates to assess ZIKV-specific or total IgG ASCs. The plates were incubated overnight at 37 °C. This was followed by incubation with biotin-conjugated anti-mouse IgG (Mabtech In) was added for 2 h at room temperature, then 100 µL/well streptavidin-ALP were added for 1 h. Plates were developed with BCIP/NBT-Plus substrate until distinct spots emerge. The plates were washed with tap water and the plates were scanned using an ImmunoSpot 4.0 analyzer and the spots were counted with ImmunoSpot software (Cellular Technology Ltd., Cleveland, OH).

### Neutralization assay

Mouse sera were first 2-fold serially diluted in DMEM with 2% FBS and 1% penicillin/streptomycin followed by incubation with mCherry ZIKV at 37 °C for 2 h. The antibody–virus complexes were then added to Vero cells in 96-well plates. At 48 h post infection, cells were visualized by fluorescence microscopy using Cytation 5 Cell Imaging Multi-Mode Reader (Biotek) to quantify the mCherry fluorescence-positive cells. The percentage of fluorescence-positive cells in the non-treated controls was set to 100%. The fluorescence-positive cells from serum-treated wells were normalized to those of non-treatment controls. A four-parameter sigmoidal (logistic) model in the software GraphPad Prism 7 was used to calculate the neutralization titers (NT_50_).

### Cytokine bioplex

Splenocytes (0.3 × 10^6^) were cultured in 96-well plates and stimulated with 1.25 × 10^4^ FFU ZIKV-FSS13025ic for 48 h, supernatants were harvested, and cytokine production was measured by using a Bio-Plex Pro Mouse Cytokine Assay (Bio-Rad).

### Statistical analysis

Survival curve comparison was performed using Prism software (GraphPad) statistical analysis, which uses the log-rank test. Values for viral load, cytokine production, and antibody and T-cell responses experiments were presented as means ± SEM. *P* values of these experiments were calculated with a non-paired Student’s *t* test.

### Reporting summary

Further information on research design is available in the Nature Research Reporting Summary linked to this article.

## Supplementary information

Supplementary Information

Reporting Summary

## Data Availability

All data generated or analyzed during this study are included in this published article (and its Supplementary Information files).
