# Supplementary material for: A genetically stable Zika virus vaccine candidate protects mice against virus infection and vertical transmission
Source: NPJ Vaccines. 2021 Feb 17;6:27. doi: 10.1038/s41541-021-00288-6 (PMC7889622; doi:10.1038/s41541-021-00288-6)
Supplement: Supplementary file 1 — Supplementary Information [file 41541_2021_288_MOESM1_ESM.pdf]

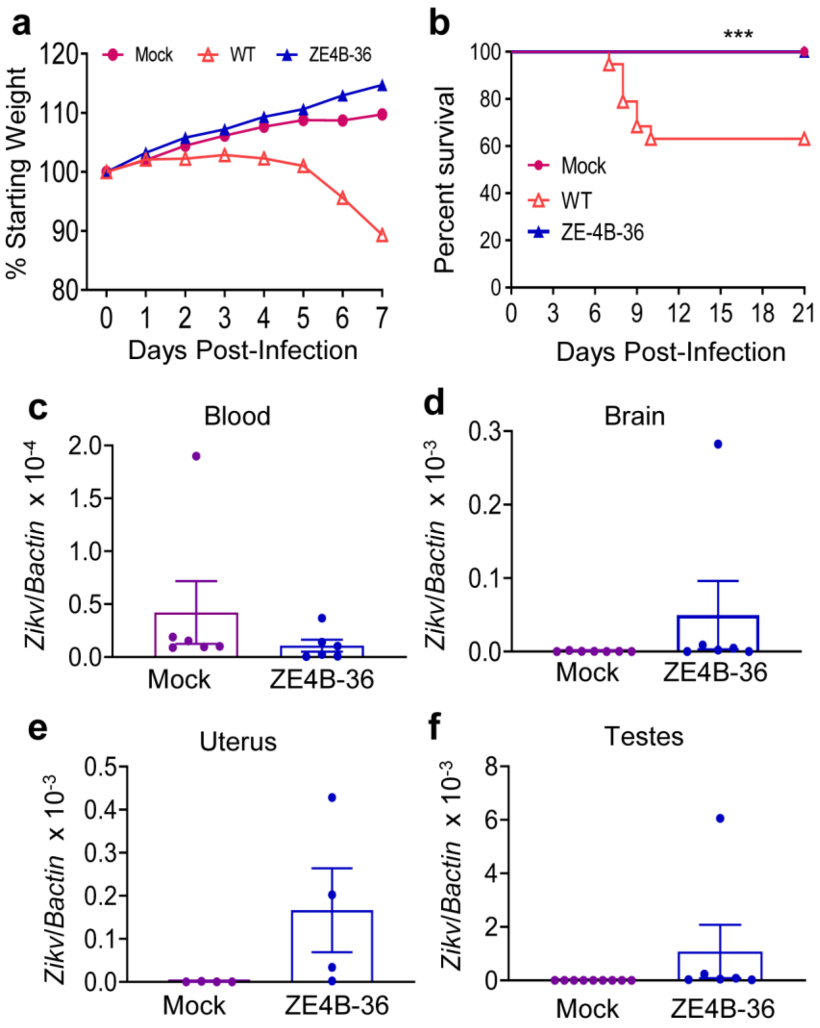

Supplementary Figure 1. ZE4B-36 mutant is attenuated and induces a brief infection in mice. **a-b**. 4-to 6-week old AB6 were infected with 1×10^4 FFU WT FSS13025ic (WT, n=19), ZE4B-36 (n=14) or PBS (mock, n=14). The immunized mice were monitored for weight loss (**a**), survival (**b**). Weight loss is indicated by percentage, using the weight on the day before immunization to define 100%. ***$P < 0.001$ compared to WT group (log rank test). **c-f.** 4 to 6-week old AB6 mice were infected with 2.2×10^5 FFU ZE4B-36 or PBS (mock). Viral loads at day 6 in blood (**c**), brain (**d**), uterus (**e**) and testes (**f**) were determined by Q-PCR assays. n= 6. Data are presented as means ± standard error of the mean (s.e.m). *** $P < 0.001$ compared to mock group (Unpaired t test).

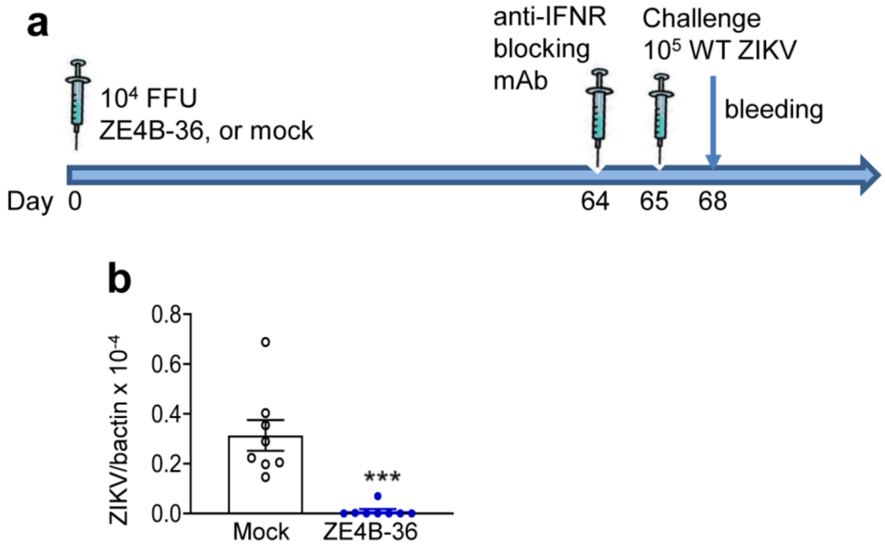

Supplementary Figure 2. Vaccination with ZE4B-36 protects WT B6 mice from ZIKV infection. 4- to 6-week old WT B6 mice were infected with ZE4B-36 or PBS (mock) subcutaneously. On day 64 pi, mice were pretreated with mouse MAR1-5A3 followed by infection with 1x 10^5 FFU ZIKV-Dakar-MA strain one day later. **a.** Schematic of vaccination and challenge. **b.** Viremia was determined by using Q-PCR at day 3 pi (n =8). Data are presented as means ± standard error of the mean (s.e.m). *** $P < 0.001$ compared to mock group (Unpaired t test).

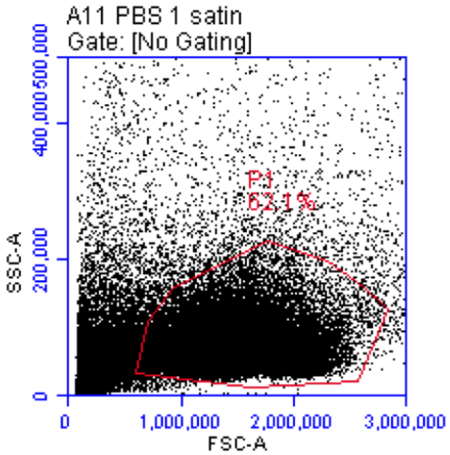

Gating Strategy: Samples were processed with a C6 Flow Cytometer instrument. Total splenocytes were gated. Dead cells were excluded on the basis of forward and side light scatter.
